# Therapeutic Outcome of Burn Patients Treated With Hyperbaric Oxygen

**DOI:** 10.7759/cureus.18671

**Published:** 2021-10-11

**Authors:** Tawfeik Alyafi, Al-Hasan H Al-Marzouki, Abdulaziz N Al Hassani

**Affiliations:** 1 Plastic and Reconstructive Surgery, National Guard Health Affairs, Jeddah, SAU; 2 College of Medicine, King Saud Bin Abdulaziz University for Health Sciences, Jeddah, SAU

**Keywords:** hyperbaric oxygen therapy, burns, carbon monoxide poisoning, thermal injury, review

## Abstract

Amongst various interventions for burns and inhalation injuries, hyperbaric oxygen therapy (HBOT) has recently been widely integrated as an adjunctive management of care due to its therapeutic properties in reducing tissue hypoxia, pathological inflammation, and augmenting neovascularization. However, the limitation of human clinical trials and data undermines its efficacy to be implemented as an adjunctive therapeutic modality in burns. The purpose of the literature review is to determine the efficacy of HBOT as an adjunct to standard management in burns. A review of the literature was done by searching PubMed, Cochrane, Medline, EMBASE, and Google Scholar for papers addressing the comparison of therapeutic outcomes between HBOT and non-HBOT in burns. Current research findings are conflicting, and the use of HBOT as an adjunct in burns management is still controversial. Therefore there is still a need for more data and research on the therapeutic benefits of HBOT in burn management.

## Introduction and background

For decades, burns and associated injuries remain one of the most prevalent burdens on patients and the healthcare system. Elevated risk of morbidity and mortality, longer hospital stay, and high cost of care are several conflicts that face the management plan of patients. As stated by the World Health Organization, burns account for an estimated 180,000 deaths annually, hence placing a significant liability on the healthcare system globally, with the vast majority in low to middle-income nations [1]. As postulated by a recent systematic review by Almarghoub et al., the overall mortality rate in burn patients of all ages in Saudi Arabia is 6.9% [2]. Mostly preventable, a burn (first, second, or third-degree) is an injury caused by exposure to extreme heat/thermal energy, radiation, contact with chemicals, electricity, and inhalation of toxic substances as carbon monoxide (CO) [1]. In addition to the thorough initial assessment/workup and universally standardized modalities of management, there has been a recent incline in the integration of hyperbaric oxygen therapy (HBOT) for the treatment of burn patients [3,4].

Reintroduced in the 1940s to treat decompression sickness in US Navy scuba divers, HBOT utilizes a special oxygen-filled chamber that’s primary goal is to utilize pure high-pressure oxygen to hasten tissue repair and restore normal body function [5]. Moreover, inhalation of high concentrations of oxygen increases the plasma partial pressure levels of oxygen (PaO2) to 20 times that of normal room air, leading to the promotion of angiogenesis, edema reduction, and immune response modulation in damaged tissues. Although HBOT is well-tolerated, certain complications could arise, such as barotrauma of the tympanic membrane, which is the most common complication, and rarely, yet worrying, hyperic-induced seizure [5,6]. Today, according to continuous evidence-based evaluation of the current literature, the Undersea Hyperbaric Medicine Society (UHMS) and European Consensus Conference on Hyperbaric Medicine (ECCHM) reported 14 indications for the use of HBOT, which includes acute thermal burns and CO poisoning, that was approved and lastly reviewed by the FDA in April 2016 (Table 1) [7,8]. However, despite approval by the FDA, HBOT in the treatment of burns remains one of the controversial practices due to the conflicting hypothesis of current literature. Therefore, recent studies have been aiming to assess the efficacy of HBOT in treating burn patients and whether to implement its use as an adjunct or a standard guideline.

**Table 1:** FDA-Approved Hyperbaric Oxygen Therapy (HBOT) Indications CO: Carbon Monoxide

FDA-Approved HBOT Indications
Air or Gas Embolism
CO Poisoning
Gas Gangrene
Crush Injury, Compartment Syndrome and Other Acute Traumatic Ischemia
Decompression Sickness
Arterial inefficiencies
Severe Anemia
Intracranial Abscess
Necrotizing Soft Tissues Infections
Refractory Osteomyelitis
Delayed Radiation Injury
Compromised Grafts and Flaps
Acute Thermal Burn Injury
Idiopathic Sudden Sensorineural Hearing Loss

According to a blinded format clinical trial conducted in Virginia Beach, USA, HBO therapeutic effects were tested on 12 healthy volunteers (seven males and five females) with an employed superficial partial-thickness UV radiation burn on the forearm. Evaluated for size, hyperemia, exudation, and epithelization of the wound for a period of six days, half of the patients (n=6) were assigned to the HBO group, and the other half (n=6) in the control group. Results showed that the HBO group showed a 42% reduction in wound hyperemia, a 35% reduction in the size of the lesion, and a 22% reduction in wound exudation reported on the second day, but no significant change in epithelization was noted [9]. On the other hand, there is a shortage of data about the effects of HBO on burns in Saudi Arabia due to the few numbers of HBO chambers scattered across the Kingdom (one in the Western Province).

As therapeutic interventions are rapidly evolving with time, HBOT is increasingly being adapted to augment the therapeutic outcome of burn patients. In addition, HBOT has the potential to reduce the healing time and fluid needs in a burn patient and increase the success rates of subsequent skin grafts. However, more studies must be conducted to implement HBOT as a standard of care in the management of burn patients.

Purpose

The aim of this review is to assess the evidence for beneficial therapeutic outcomes in burn patients who underwent HBOT in comparison to patients who did not.

Methods

**Figure 1:**
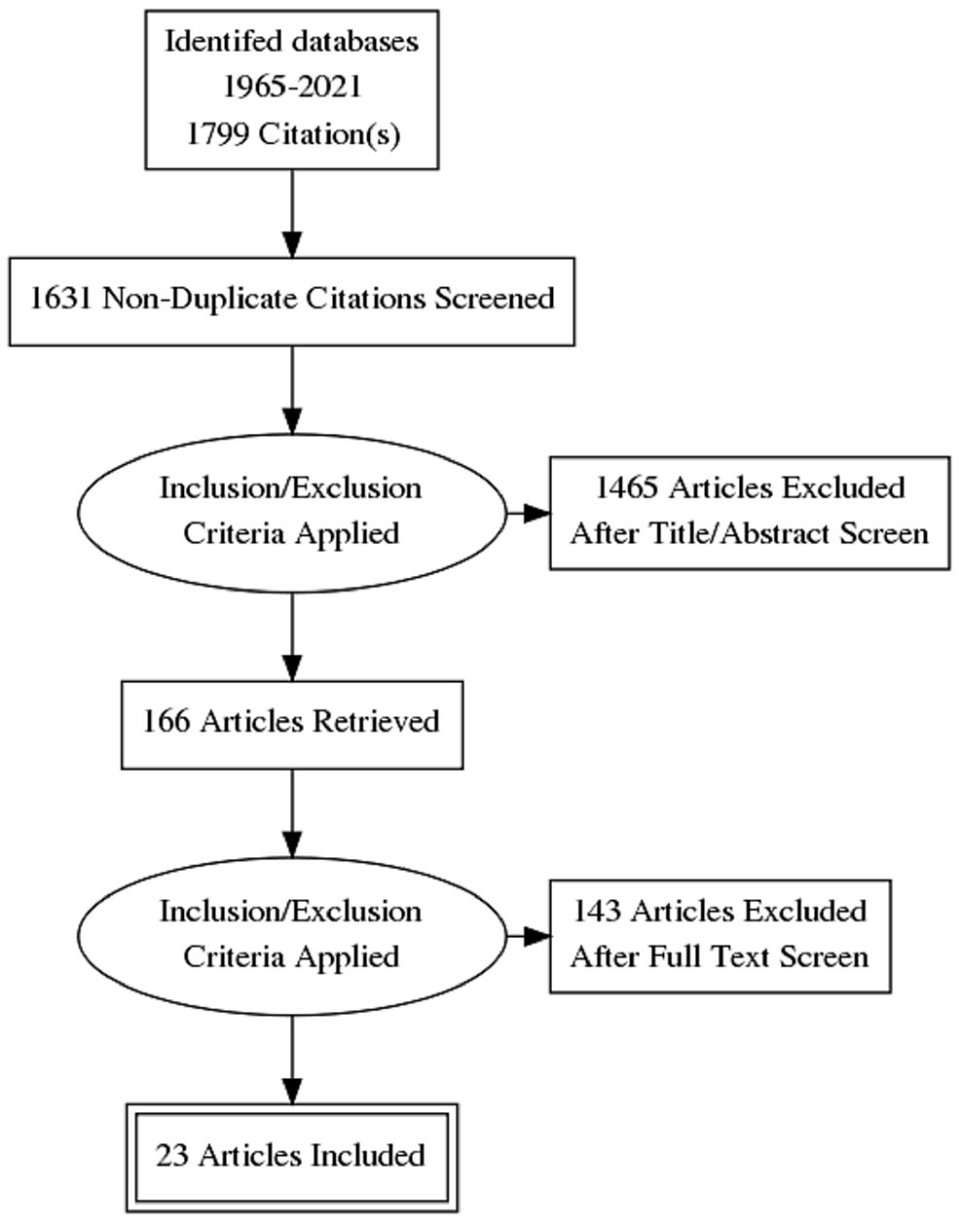
Flow Chart Describing Article Selection and Screening Process

A review of the literature was conducted independently by two authors by searching PubMed, Cochrane, Medline, EMBASE, and Google Scholar to identify all relevant systematic reviews, with or without meta-analysis, randomized controlled trials, and other original research. A variety of keywords and Medical Subject Headings (MeSH) terms were used, including “hyperbaric oxygen therapy,” “hyperbaric oxygenation,” “burns,” and “thermal injuries” to decrease the possibility of missing any related publication. Titles and abstracts of all the results were examined and reviewed. Duplicate articles were excluded. Publications addressing the comparison of therapeutic outcomes between HBOT and non-HBOT in burns were then reviewed in full text, as well as the bibliographies for any additional relevant studies. A total of 23 relevant publications ranging from 1967-2021 that included both human and animal studies were filtered and included. The restrictions applied to the search were excluding all non-English publications and irrelevant articles to our title (Figure 1).

## Review

While the use of HBOT in treating and enhancing burn wounds and skin grafts proved to be effective in animal models, human experiments are still scanty. An experiment on rabbits with second-degree burns conducted by Hatibie et al. revealed that HBOT improves healing by increasing migration of inflammatory cells and re-epithelization [10]. A different experiment by Dinar et al. studied the effect of HBOT on fibrovascular ingrowth of porous polyethylene blocks implanted under burn scar tissue. It found that HBOT improved collagen synthesis and neovascularization, and consequently enhanced biointegration of porous polyethylene in burn scars [11]. In addition to effects on burn wound healing, some experiments proved beneficial outcomes in using HBOT on grafts in animals. For example, Fodor et al. found that the survival of composite grafts in rats improved after being placed in a hyperbaric chamber for 90 minutes daily for two weeks. This improvement was especially prominent on the inner surface of the graft [12]. In the same way, HBOT proved to promote faster healing and epithelialization of second-degree wounds in the experiments of Korn done on guinea pigs [13]. Additionally, an experimental study by Türkaslan et al. assessed the impact of HBOT in recovering the zone of stasis in rats, demonstrated a positive effect at the cellular level of rats in the first 24 hours, decreased edema, and augmented neovascularization within five days [14].

On the other hand, data on the use of HBOT in human burns are still inconclusive, and current literature is often contradictory. A matched controlled study by Waisbren and colleagues included 36 patients who received HBOT and matched 36 controls found that mortality, mean day of death, and hospital time were similar in both groups. On top of that, they reported increased kidney damage and sepsis in the HBOT group. The researchers attributed these findings to the possibility of the presence of an undetected selection factor rather than being due to the HBOT itself [15]. Similarly, a review by Leach et al. suggests that the use of HBOT in usual thermal burns is not beneficial as there is no convincing evidence to support its use. The same review reports that the hospital stay, mortality, and need for autografting are virtually identical in both treatments [16]. In contrast, the experiments of Hart et al. in 1974 and Merola et al. in 1978 favored the use of HBOT in burn wounds as its use accelerated healing and reduced mortality [17,18]. Furthermore, Niezgoda et al. noted that patients who underwent HBOT following receiving superficial burn wounds had a 42% reduction in wound hyperemia, 35% in size of the lesion, and 22% in wound exudation when compared with volunteers not receiving HBOT [9].

Burn injuries can cause significant painful wounds that alter the normal protective physiology of the skin, as well as affecting the psychological well-being of burned patients. To address these issues, skin grafts are being used extensively in burn patients to cover burned areas. In 1967, a randomized controlled trial (RCT) by Perrins et al. showed an increase in graft survival. The same study reported a graft take of 64% in the group treated with HBOT, as opposed to the 17% take in those not treated with HBOT [19]. Likewise, Rasmussen et al. conducted an RCT to assess antinociceptive effects of HBOT induced by thermal injuries in otherwise healthy humans. The thermal injury was associated with a significant decrease of pinprick thresholds and heat pain threshold. Also, there was no difference in primary hyperalgesia areas between those treated with HBOT and those who were not. On the other hand, attenuated secondary hyperalgesia response was observed in those treated with HBOT [20].

The high rate of morbidity and mortality in burns has yielded poor outcomes in patients of all age groups. As stated by Guldogan et al., the mortality rate after thermal burns (>30% total body surface area [TBSA]) was the highest of any burn type at 44.2% of 224 patients [21]. The use of adjunctive HBOT in burns has sought to determine its significance to reduce morbidity and mortality rates effectively. As concluded by Cianci and Sato in the literature review, multiple studies have displayed a significant reduction in the mortality rate of burn patients in comparison to the non-HBOT controls [22]. An RCT by Grossman postulated that burn patients treated with HBO displayed a reduced mortality rate to those who did not (41% of 24 patients in the HBOT group vs. 68% of 25 patients in the non-HBOT group) [23]. Moreover, Struzyna et al. has demonstrated a similar conclusion in multiple clinical trials that concluded an improvement in mortality rates after the use of HBOT in comparison to the non-HBO treated group. Nevertheless, they also portray other contradicting trials that show insignificant and insufficient differences in the rates between the two groups [24]. Similarly, regarding morbidity and mortality, Weitgasser et al. and Villanueva et al. reviews have reported no clinical difference between the HBOT and non-HBOT groups [7,25]. Hence, there is insufficient data to support or refute the influence of HBOT in decreasing morbidity and mortality rates.

Thermal burns cause high morbidity and mortality, prolonged hospital stay, disability, limitations in performing daily activities, and not to mention the generation of a high cost of care per patient. Length of hospital stay (LOS) in burn patients is influenced by various factors such as TBSA percentage, burn and wound type, and the presence of concurrent infection or inhalational injury. According to a prospective study by Abdelwahab et al., the LOS demonstrated a direct positive correlation with morbidity and mortality rates in acute burns. Moreover, out of the 82 patients in the study, the mean age of patients was 16.5 years, mean LOS was 24.23 days, and mortality rate was 9.8% [26]. On the other hand, as concluded by a 20-patient RCT conducted by Oley et al., a significant decrease in hospital stay was reported in patients receiving HBOT (17.5 days, 4.3%) to the non-HBOT control group (26.3 days, 7.6%) [27]. Similarly, Cianci’s literature review has reported a reduction in the hospital stay duration in burn patients subjected to adjunctive HBOT [22]. A trial conducted by Grossman reported a decrease in hospital stay in patients treated with HBO dependent on TBSA percentage [23]. However, a trial by Chiang et al. reached the conclusion of an insignificant LOS between the HBOT and non-HBOT group, whether in the ICU (HBOT vs. control: 18.11 +/- 4.41 vs. 33.33 +/-14.13 days, p = 0.318) or hospital wards (HBOT vs. control: 77.92 +/- 6.61 vs. 70.21 +/-16.23 days, p = 0.600) [28]. In addition, a literature review published by Struzyna et al. demonstrated multiple studies that concluded no significant difference in LOS between patients treated with HBOT to those who were not [24]. In regard to cost-effectiveness, two independent literature reviews by Cianci and Struzyna concluded that HBOT in burns decreases the overall cost of therapy, and in the first review, a saving cost of $107,000 (36%) per patient was reported [22,24]. 

## Conclusions

All in all, since the 1960s, HBOT has been used to treat a wide variety of conditions, either as a standard regimen in the management plan or as an adjunctive treatment. Nevertheless, the use of HBOT as an adjunct to treating thermal burns remains controversial due to limited and conflicting evidence. The urge for a higher quality and quantity of RCTs of the use of HBOT in treating burns is essential to further understand and assess the harms and benefits, and determine whether to instill HBOT as an adjunct to the standard course of management in such patients or not. In the meantime, if a clinician opts to use HBOT in addition to the standard burn care, it should be used with extreme caution to minimize any possible harm to the patient.
